# High-throughput genotyping of a common deletion polymorphism disrupting the TRY6 gene and its association with breast cancer risk

**DOI:** 10.1186/1471-2156-8-41

**Published:** 2007-06-29

**Authors:** Kerstin Wagner, Ewa Grzybowska, Dorota Butkiewicz, Jolanta Pamula-Pilat, Wioletta Pekala, Karolina Tecza, Kari Hemminki, Asta Försti

**Affiliations:** 1 Division of Molecular Genetic Epidemiology, German Cancer Research Center (DKFZ), Im Neuenheimer Feld 580, 69120 Heidelberg, Germany; 2 Department of Molecular Biology, Center of Oncology, Maria Sklodowska-Curie Institute, Gliwice, Poland; 3 Center for Family and Community Medicine, Karolinska Institute, Huddinge, Sweden

## Abstract

**Background:**

Copy number polymorphisms caused by genomic rearrangements like deletions, make a significant contribution to the genomic differences between two individuals and may add to disease predisposition. Therefore, genotyping of such deletion polymorphisms in case-control studies could give important insights into risk associations.

**Results:**

We mapped the breakpoints and developed a fluorescent fragment analysis for a deletion disrupting the *TRY6 *gene to exemplify a quick and cheap genotyping approach for such structural variants. We showed that the deletion is larger than predicted and encompasses also the pseudogene *TRY5*. We performed a case-control study to test an association of the *TRY6 *deletion polymorphism with breast cancer using a single nucleotide polymorphism which is in 100% linkage disequilibrium with the deletion. We did not observe an effect of the deletion on breast cancer risk (OR 1.05, 95% CI 0.71–1.56).

**Conclusion:**

Although we did not observe an association between the *TRY6 *deletion polymorphism and breast cancer risk, the identification and investigation of further deletions using the present approach may help to elucidate their effect on disease susceptibility.

## Background

An unexpectedly large amount of structural variation in the human genome has recently become apparent [1]. The identification of genomic deletions, insertions and inversions, ranging from 1 kb to several Mb in size, has broadened our knowledge about genetic variation between individuals, which may lead to new insights into phenotypic variation and clinical outcome [2]. The rearrangements can encompass exons or even entire genes and their regulatory regions. Such structural variants, called copy number polymorphisms (CNPs), have been identified by array-based methods [3-6]. Interestingly, a high percentage of copy number variants has been found to be in close proximity to segmental duplications, suggesting a recombination mechanism between the repetitive sequences [4,7,8]. Owing to the high sequence similarity between duplications and their consequent high recombination potential, segmental duplications are frequently found at breakpoints of both disease-associated and evolutionary rearrangements [9]. The HapMap data on family trios have been used to identify CNPs [10,11]. As deletions normally are not detected by standard single nucleotide polymorphism (SNP) genotyping methods, hemizygotes are usually misclassified as homozygotes. When a deletion is transmitted from parent to offspring, the child will show a null genotype or a genotype violating the rules of mendelian inheritance [10]. However, the exact borders of the structural variants still remain to be ascertained, which makes it difficult to perform high-throughput genotyping of CNPs. Furthermore, linkage disequilibrium (LD) between deletions and SNPs has been demonstrated [11,12], suggesting that these variants show a similar evolutionary history [12]. The use of SNP genotyping may also be an alternative for the genotyping of the deletions. However, recent reports have raised concern over the use of SNPs as surrogates for CNPs in structurally dynamic regions [7,13].

**Figure 1:**
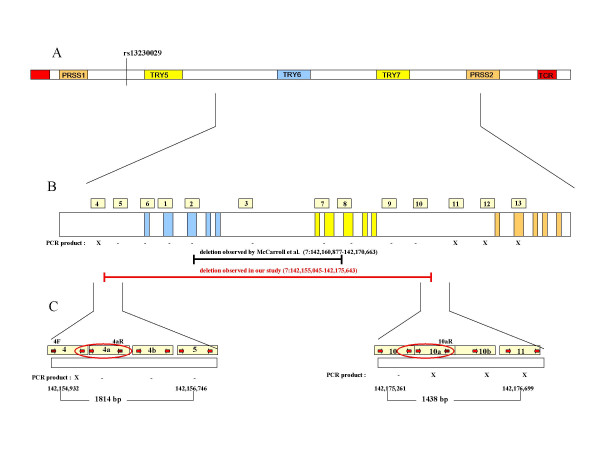
(A) Schematic organisation of the human T-cell receptor locus on chromosome 7 containing the trypsinogen genes *PRSS1, PRSS2 *and *TRY6 *and the pseudogenes *TRY5 *and *TRY7*. The figure is not drawn to scale. Numbering according to NT_007914.14, NCBI build 36. (B) Magnification of the region investigated to map the breakpoints. The PCR fragments used to map the deletion are numbered 1–13, corresponding to the primer pairs shown in Table 1. The fragments for which a PCR product was obtained in the samples homozygous for the deletion is indicated with X; – indicates no PCR product in the homozygote samples. The deletion reported by McCaroll et al. [11] is indicated as well as the revised deletion size. (C) Fine-scaling of the breakpoints and cut-out of the fragments used to narrow down the breakpoint region. The breakpoints are marked with a red circle.

As CNPs contribute to the genetic variability between two individuals, the identification of their influence on disease association remains a great challenge. We developed a simple PCR-based assay to genotype exemplarily a common deletion polymorphism encompassing the trypsinogen C (*TRY6*) gene [11,14]. *TRY6 *is a member of a highly homologous serine protease family (PRSS) which comprises the active trypsinogen genes 1 (alias *PRSS1, TRY1 *or *T4*), 2 (alias *PRSS2, TRY2 *or *T8*) and 4 (alias *PRSS3, TRY4 *or *T9*), the transcribed pseudogene *TRY6 *and the pseudogenes *TRY5 *and *TRY7*. These genes cluster within the human T cell receptor (TCR) locus on chromosome 7 (Figure 1A) [14]. They exhibit ~91% overall similarity at both nucleotide and protein levels and harbour numerous interspersed repeats. The trypsinogen locus is of dynamic nature. It has been shown that, in the course of evolution, *PRSS3 *has duplicated and translocated from chromosome 7 to chromosome 9. Thus, the locus may be a subject of frequent recombination events. Trypsinogens are the precursors of the serine protease trypsin that has been linked to tumour progression in colorectal cancer through activation of matrix metalloproteinases, which degrade extracellular matrix components [15]. Additionally, TRY6 has been shown to be overexpressed in metastasising non-small cell lung tumours, correlating with survival [16].

We present an approach to specify the breakpoints of the common deletion polymorphism encompassing the *TRY6 *gene and then describe a genotyping assay, using fluorescent multiplex PCR, to investigate whether the deletion is associated with breast cancer risk. Alternatively, the deletion can be genotyped by analysing a neighbouring SNP which is in 100% LD with the deletion polymorphism. These methods can be adapted for other deletion polymorphisms.

## Results

### Identification of TRY6 breakpoints

**Figure 2:**
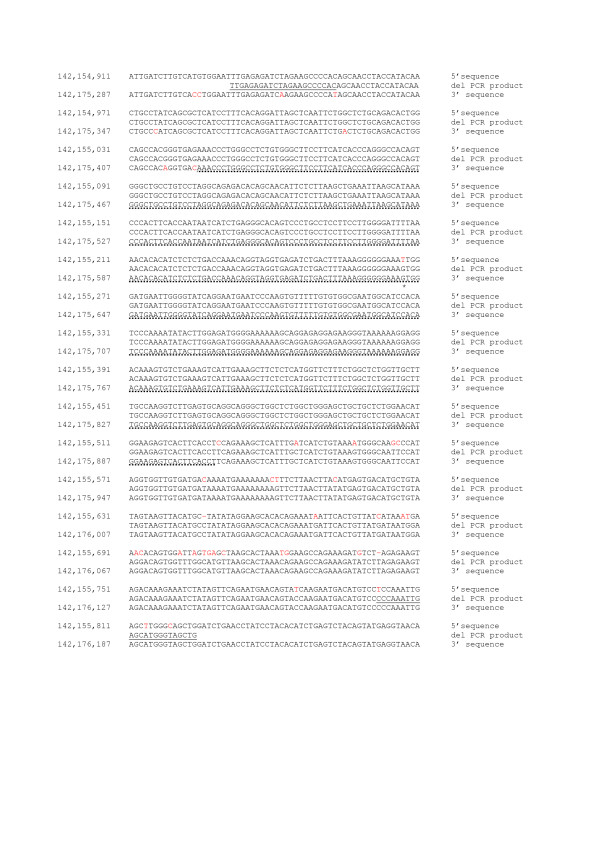
Sequence alignment of the deletion-specific *TRY6 *PCR product (generated with 4F and 10aR primers) sequence and the respective 5' and 3' sequences of the full-length *TRY6*. The 5' and 3' *TRY6 *sequences were derived from Genbank NT_007914.14 (NCBI Build 36). PCR primer sequences are underlined. Letters in black indicate identity, letters in red indicate mismatches; points (·) underscore the homologous deletion breakpoint region. * shows the position of discrepancy between our sequencing results and the GenBank sequence (NT_007914.14)

The *TRY 6 *deletion has been described by McCarroll et al. to range at least from 142,160,877 to 142,170,663 on chromosome 7 (NCBI build 36), spanning a minimum region of 9786 bp [11]. We designed primers that flank the breakpoints of the predicted deletion (fragments 2 and 8, Figure 1B) and primers within the deletion (fragments 3 and 7) to confirm the known homozygous carriers among the CEPH individuals. Homozygotes for the deletion should not give a PCR product within the deleted region. Additionally, we used primers for an independent gene fragment (growth hormone releasing hormone, GHRH) to show that the samples could be amplified. No PCR products were observed for the fragments 2, 3, 7 and 8 in samples from the homozygous individuals, indicating that the deletion was longer than predicted. We designed new primers outwards in intervals of 1.5–2 kb. For homozygous samples, we did not obtain a PCR product until the fragments 4 and 11 (Figure 1B). To map the breakpoints, we designed primers surrounding the presumed region and covering the complete region between fragments 4 to 5 and 10 to 11, respectively (Figure 1C). The deleted region mapped between nucleotides 142,155,045 and 142,175,904 (Figure 2). Sequencing of a homozygous sample showed a sequence as outlined in Figure 2. The region is flanked by two 484 bp long sequences that differ in only one nucleotide (T/G) after the first 222 bp. In the homozygous samples this sequence was present only once, suggesting that the deletion is a result of a non-allelic homologous recombination between these two sequences. Furthermore, we identified two discrepancies to the published sequence in the NCBI database (NT_007914.14, Build 36) by comparing the sequences of the full-length and the deletion-containing samples. At 142,155,267, a T instead of the reported G constitutes the wild type sequence and at 142,175,643 a G is present instead of the reported T. Since the sequences are nearly 100% identical, these initial sequencing errors could be due to unspecific primers; and both positions are reported to be polymorphic (rs4019213 and rs2734212, respectively). Search with the RepeatMasker database revealed no retroviral elements within the breakpoint region. In contrast to the reported 10 kb size of the deletion, we enlarged the disrupted region to about 20.6 kb.

### Fragment analysis for a case-control study

**Figure 3:**
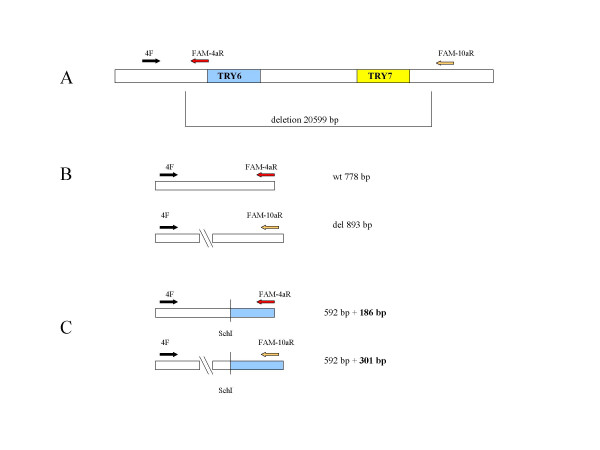
(A) Schematic diagram of the *TRY6 *deletion and the location of the primers used for fluorescent fragment analysis. (B) PCR products amplified by multiplex PCR. (C) PCR products created for fluorescent fragment analysis by SchI digestion.

**Figure 4:**
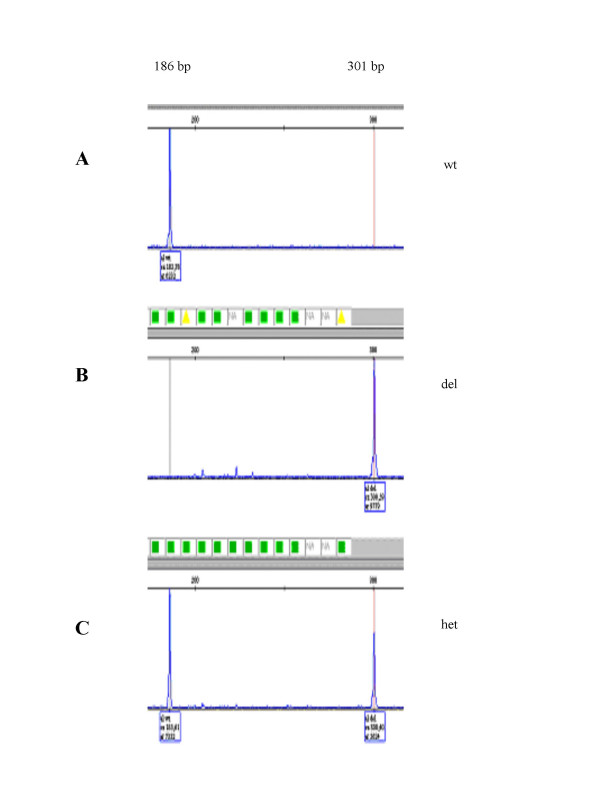
An example of the fragment analysis results using the GeneMapper 4 software. (A) A homozygous sample for the wild type allele, 186 bp. (B) A homozygous sample for the deletion allele, 301 bp. (C) A heterozygous samples, 186 bp + 301 bp.

In order to genotype the deletion in a high-throughput process, we developed a fluorescent fragment analysis assay using a triplex PCR with FAM-labelled reverse primers. The forward primer was located 5' to the breakpoint (primer 4F), the reverse primer for detecting the wild type allele within the deletion sequence (primer 4aR) and the reverse primer for detecting the deletion 3' to the breakpoint (primer 10aR) (Figure 3). This enabled us to specifically amplify both the wild type and the deletion alleles, even in heterozygous samples. The high sequence homology within the trypsinogen gene cluster and the length of the repeat sequence at the breakpoints (484 bp) limited the options for primer selection, thus the resulting specific fragments for the wild type and the deletion alleles were 778 bp and 893 bp, respectively. Since these fragments were too large to run on the Genetic Analyzer efficiently, we digested the PCR product with *Sch*I. The resulting fragments of 186 bp and 301 bp were visualised after the fragment analysis run (Figure 4) and called automatically by the GeneMapper software.

### rs13230029 is not associated with breast cancer risk

Common deletions and nearby SNPs have been shown to be in strong LD [11,12]. We screened several SNPs surrounding the deletion as described by McCarroll et al. [11] in a small set of 23 samples in order to confirm the LD between the SNPs and the deletion. We chose a SNP 8240 bp upstream of the deleted region (rs13230029, Figure 1A) to perform a case-control study with 397 Polish familial and early age breast cancer cases and 454 regionally and ethnically matched unrelated female controls using TaqMan allelic discrimination. The genotype distribution followed Hardy-Weinberg equilibrium. There were no differences in the allele and genotype frequencies between the cases and the controls (Table 2). We carried out a fragment analysis on 200 samples and confirmed that the SNP was completely linked with the deletion. We conclude that the deletion polymorphism of *TRY6 *is not associated with breast cancer risk.

**Table 1:** Primer sequences used to map the breakpoints.

**Primer combination**	**Primer sequence**	**Product size (bp)**
1F/R*	F: GAC ACA AAG ACG TGG GAG TC R: GAG GCA GCC TGG CTG GGA	384
2F/R*	F: GTG AAA GAG GCT GGG AAG GTG R: CCC TTC TTT CAC AGC TGG GGC	249
3F/R*	F: ACA GGT GAT AAA AGC CCG AGC C R: GCC CTC AGA TGA TTA TTG GTG AAG T	144
**4F**^†^**/R**	F: TTG AGA GAT CTA GAA GCC CCA C R: GCC CTC AGA TGA TTA TTG GTG AAG T	247
5F/R*	F: TAA GTC TCC TTT TAG ATG CCA CC R: GGA GTT TTC ATG TAA GCA GCA GTG	261
6F/R*	F: CTC CAG AGC TAT AAA GAC GGG C R: CGG CAG GGC ATA TGT CTG CCA	159
7F/R*	F: AAA AGA GAG AAG CAT TCA GTG GG R: CGG TTG CTT CCT GCT AAT TAG AA	261
8F/R*	F: GAA TGA ACA GTT CAT CTA TGC G R: CGG TGT GCT TCG TTC TGG AAA T	272
9F/R*	F: TCT GAG GGC TGT GAC ACC ATG R: GAC ACA GGT GAC ATG AAG CCT G	100
**10F/R**	F: CTG GGA AAG GAT CCC TCA AG R: GTC AAC TGT GGC TGC CAG TG	160
**11F/R**	F: ACA TGT TCA GGG ACA ACA CAG C R: CAC ACC TCT CTG CTC ATG AAT AA	97
**12F/R**	F: AAC TCT GAC ATG TGA TCA GGG G R: CAT GTG CAT CCT GTC ATA GGT TC	143
**13F/R**	F: AAG CAG CCA CAG GCT GGG AGC R: CCT GGC TGG GAG TTT TGC AGT	273
**4aF/R**^†^	F: GAT GAA TTG GGG TAT CAG GAA TG R: GCT CAC TAA TCC ACT GTG TTT CAT	437
4bF/R*	F: CTG TGA TTG TTT AAG GAA GAG CG R: CAC AAC TCT CTG CTC ACA GAT AA	380
**10aF/R**^†^	F: CCC AAG TGT TTT TGT GTG GCG AAT GG R: CAG CTA CCC ATG CTC AAT TTG G	530
**10bF/R**	F: GGA AGG ACA GTG GTT TGG CAT G R: GTC AAC TGT GGC TGC CAG TG	620

* fragment is within the deletion region

^† ^primers used for triplex PCR

**Table 2:** Allele and genotype frequencies, odds ratios, 95% confidence intervals and p-values for the SNP rs13230029.

**rs13230029**	**Cases (%)**	**Controls (%)**	**OR (95% CI)**	**p-value**
**CC**	136 (0.34)	159 (0.35)	1	
**CG**	190 (0.48)	216 (0.48)	1.03 (0.76–1.39)	0.86
**GG**	71 (0.18)	79 (0.17)	1.05 (0.71–1.56)	0.81
**G%**	0.42	0.41		

## Discussion

Large genomic variations show a potential at least as great as SNPs to have an impact on disease susceptibility, given that regulatory regions, exons and even whole genes can be deleted, duplicated or disrupted [2]. Deletions result from non-allelic homologous recombination events that occur between blocks of duplicated sequences [4,7]. Recent studies have provided a preliminary characterisation of deletions and paved the way to disease association studies [3-6,10-12]. However, fast, cheap and reliable techniques are needed to perform case-control studies for the identification of disease correlation.

Real-time PCR can be used for the detection of individual deletions or duplications but it is not appropriate for multiplexing [2]. Furthermore, the optimisation process takes time and the throughput is limited. For the amplification of multiple regions, quantitative multiplex PCR of short fluorescent fragments (QMPSF), multiplex amplifiable probe hybridisation (MAPH) and multiplex ligation-dependent probe amplification (MLPA) are more adequate [2]. However, MAPH and MLPA require several handling steps, the commercially available kits are still expensive and pre-designed for only common sites and these methods are not suitable for the identification of heterozygous genotypes. QMPSF is a faster method, yet the optimisation, especially for heterozygotes, is time-consuming and tedious and one should have control samples with known genotypes for comparison of the results. All the above described methods could be useful for identifying breakpoints in homozygous samples where it is only important to know if the product is present or absent. A quicker and safer approach is a triplex PCR using primers that flank the deletion break points, as described here and elsewhere [3,17,18]. If the primers are labelled fluorescently, an automatic analysis ensures a fast evaluation of the results. However, it is essential to know the deletion borders to design the primers.

We refined the borders of the deletion by amplifying short fragments in intervals of 1500–2000 bp throughout the predicted and the extended region of the *TRY6 *gene deletion polymorphism in individuals with known homozygous genotypes obtained from the HapMap subjects [11]. Homozygote carriers showed the absence of the PCR products, while the fragments were present in the wild type and the heterozygote samples. As an internal control for the sample quality, we simultaneously amplified a fragment of an independent gene. We mapped the breakpoints by sequencing the complete region between the two fragments flanking the deletion in the homozygous samples and by comparing the obtained sequence to the published one (NT_007914.14, NCBI Build 36). A triplex PCR with primers neighbouring the borders enabled discrimination between the three genotypes. Due to the fluorescence of the reverse primer, the products could be visualised on a sequencer and analysed automatically with the appropriate software. This method is fast, cheap (0.70€/genotype) and easy to perform.

*TRY 6 *is deleted in a common polymorphism [11,14]. Although this locus appears to encode a protein similar to trypsinogen, the locus is thought to be a transcribed pseudogene [19]. ESTs support its transcription, but expression of its predicted protein has not been observed. The predicted protein sequence of TRY 6 differs significantly from the known functional trypsinogens, including a different amino acid at the conserved residue 122 which is important for autolysis. However, it has been suggested that it is expressed in minute amounts in the thymus [14]. A recent study has identified TRY6 to be up-regulated in metastatic non-small cell lung cancer leading to an enhanced cell migration [16]. Patients with high expression of TRY6 also had a substantially worse prognosis than patients without TRY6 expression. We picked this deletion polymorphism to test our method because it was shown to be real, frequent and affecting the coding region [11]. We did not find an association of the *TRY6 *deletion with breast cancer susceptibility. With our sample size, considering that the cases had familial or early onset breast cancer and that ~17% of the controls were homozygous for the deletion, we had an >80% power to detect an OR of 0.6. Thus, we can only exclude a major to moderate effect of the *TRY6 *deletion on breast cancer risk. The negative findings of our study may be explained by the fact that *TRY6*, and the deleted neighbouring gene, *TRY5*, are pseudogenes. Although *TRY6 *mRNA expression has been established, the function of the gene remains to be clarified.

Several reports have shown that deletion polymorphisms show strong LD with common SNPs [11,12,18]. We genotyped a SNP and confirmed its LD with the deletion. Genotyping a SNP is faster and cheaper than detecting the structural variant. However, recent studies have found only modest evidence of LD between CNPs and HapMap SNPs, suggesting that duplication rich regions are not suitable for the identification of linked SNPs due to technical restrictions [7,13]. This raises the concern that several genomic regions most prone to rearrangements have inadequate SNP density to successfully map CNPs with the use of available SNP markers. Thus, additional methods, such as the fluorescent fragment analysis for deletion polymorphisms described by us, are needed to reliably genotype CNPs.

The comparability of the results generated from various array-based studies is currently the major obstacle in the field, contributing to confusion in data interpretation between different reports. Because accurate genotyping requires exact sequence data, refining the deletion breakpoints should rank first. Furthermore, the mapping of CNP breakpoints will also enable the identification of nearby SNPs, which would be in LD with the deletion. Thus, the description of all variants will help to design studies relating to disease, pharmacogenomics and clinical practice. They can be searched in the Database of Genomics Variants [20], which is regularly updated with the latest detected CNPs. The development of methods to identify other subtle variations such as inversions or insertions still remains a challenge.

We presented a simple high-throughput approach to genotype common deletion polymorphisms. Although we did not observe an association between the *TRY6 *deletion polymorphism and breast cancer risk, the identification and investigation of further deletions using the present approach may help to elucidate their effect on disease susceptibility.

## Methods

### Breakpoint mapping

Based on the analysis carried out on the Hapmap samples by McCarroll et al. [11,21], we selected CEPH individuals homozygous for the *TRY6 *deletion polymorphism to define the breakpoints of the deletion. We amplified several fragments throughout the *TRY6 *gene region (Figure 1B). The fragments were first limited to the predicted region but then extended outwards on both directions. Homozygotes for the deletion should not give a PCR product within the deleted region, which was used in mapping the breakpoint. The primer sequences and product sizes are listed in Table 1. Amplification was performed with 5 ng genomic DNA in a 10 μl reaction volume using 1× PCR buffer, 1.5 mM MgCl_2_, 0.11 μM dNTP Mixture (Invitrogen, Paisley, UK), 0.15 μM of each primer (Thermo Electron Corporation, Ulm, Germany) and 0.3 U PlatinumTaq Polymerase (Invitrogen). The PCR was carried out in a GeneAmp 9700 PCR system (Applied Biosystems, Foster City, USA) using 94°C for 2 min, followed by 3 cycles of 94°C for 1 min, 67°C for 1 min and 72°C for 1 min and 32 cycles of 94°C for 30 s, 66°C for 30 s and 72°C for 30 s. The final extension was performed for 6 min at 72°C. The PCR products were visualised on 1.5% agarose gels stained with ethidium bromide.

### DNA sequencing

The PCR product was cleaned-up using 0.75 μl ExoSapIT (USB Amersham, Uppsala, Sweden) for 40 min at 37°C followed by 15 min at 85°C. The sequencing reaction was carried out using the BigDye Terminator Cycle Sequencing Ready Reaction Kit (Applied Biosystems) on the Genetic Analyzer 3130XL as described earlier[22] The original data was analysed by the Sequencing Analysis 5.2 and DNASTAR Lasergene 5.0 (DNASTAR Inc., Madison, USA) softwares.

### Sequence alignment

The sequence of a sample with a homozygous deletion was aligned to the sequence surrounding the observed breakpoint on both sides. The reference sequence was derived from Genbank NT_007914.14, NCBI build 36. The alignment (Figure 2) was performed using MegAlign 5.05 (DNAStar Inc., USA).

### Subjects

The analysis of the *TRY6 *deletion was performed on genomic DNA from 397 Polish familial and early age breast cancer cases (mean age 46 years, range 26–81) and 454 regionally and ethnically matched unrelated female controls (mean age 42 years, range 16–79). The inclusion criteria for the cases were (i) at least 2 first-degree relatives with breast and/or ovarian cancer regardless of age, (ii) breast cancer diagnosed below the age of 35 without family history, (iii) bilateral breast cancer regardless of the family history, (iv) breast and ovarian cancer diagnosed in one patient regardless of the family history and (v) breast cancer diagnosed below 50 years of age regardless of family history[23] The subjects corresponding to criteria (i)-(iv), 215 cases, were collected during the years 1997 to 2002 by the Chemotherapy Clinics and the Genetic Counselling Service (Gliwice, Poland) and the subjects corresponding to criteria (v), 182 cases, were collected during the time period December 2002 to March 2004 by the Surgery Clinics (Gliwice, Poland). The cases were tested for four founder mutations in *BRCA1 *and two in *BRCA2*, which account for ~90% of the mutations in the Polish population, and found to be negative [24]. We included familial and early onset cases to our study because it has been shown that selection of cases based on the family history increases the power to detect low-penetrance variants [25,26]. The study was approved by the ethical committee of the University of Heidelberg.

### Fluorescent fragment analysis

In order to genotype individuals for the deletion polymorphism (Figure 3), we used a triplex PCR with the primers 4F, 4aR and 10aR (Table 1). The reverse primers were labelled with 6-FAM (Invitrogen). We performed the amplification with the conditions mentioned above. The resulting product was digested with 4 U *Sch*I (Fermentas, St. Leon-Roth, Germany) for 4 h at 37°C. 1 μl of the digestion product was added to 10 μl HIDI-Formamide/GeneScan ROX350 standard size marker (mixed according to the manufacturer's instruction, Applied Biosystems). The mixture was denatured for 5 min at 95°C, loaded onto the ABI PRISM 3130XL Genetic Analyser and analysed by the GeneMapper software version 4.0 (Applied Biosystems).

### TaqMan analysis

The TaqMan assay for SNP rs13230029 was obtained as custom assay from Applied Biosystems and the assay details are available on request. The reaction was performed in 5 μl using 5 ng of genomic DNA, 1× TaqMan Universal Master Mix (Applied Biosystems) and 0.6× Assay-Mix (40×) per reaction. PCR was performed at 50°C for 2 min, 95°C for 10 min and 35–45 cycles at 92°C for 15 s and 60°C for 1 min. PCR was performed in a GeneAmp PCR System 9700 thermocycler and the number of cycles was dependent on the genotype clustering. The samples were read and analysed in an ABI Prism 7900 HT sequence detection system using the SDS 1.2 software (Applied Biosystems).

### Statistical analysis

The observed genotype frequencies in the breast cancer cases and controls were tested for Hardy-Weinberg equilibrium (HWE) and the difference between the observed and expected frequencies was tested for significance using the χ^2^-test. Odds ratios (ORs) and 95% confidence intervals (95% CIs) were calculated for associations between genotypes and breast cancer. The calculations were carried out using the HWE test tool offered by the Institute of Human Genetics, TU Munich [27]. Power calculation was carried out using the PS software for power and sample size calculation [28,29].

## Authors' contributions

KW participated to study design, carried out the laboratory analyses and drafted the manuscript. EG was responsible for the study cohort. DB, JP-P, WP and KT participated in the sample collection and carried out the BRCA1/2 mutation screening. KH participated to study design and was involved in manuscript preparation. AF participated to study design, was responsible for supervising laboratory work and was involved in manuscript preparation. All authors read and approved the final manuscript.
